# Cognitive function in metformin exposed children, born to mothers with PCOS – follow-up of an RCT

**DOI:** 10.1186/s12887-020-1960-2

**Published:** 2020-02-10

**Authors:** Hanne Klæboe Greger, Liv Guro Engen Hanem, Heidi Furre Østgård, Eszter Vanky

**Affiliations:** 1 Departement of Mental Health, Faculty of Medicine and Health Sciences, NTNU -Norwegian University of Science and Technology, Trondheim, Norway; 2 St.Olavs Hospital, Trondheim, Norway; 3 Department of Clinical and Molecular Medicine, Faculty of Medicine and Health Sciences, NTNU -Norwegian University of Science and Technology, Trondheim, Norway

**Keywords:** Metformin, PCOS, Offspring cognitive function, Androgen

## Abstract

**Background:**

Metformin is widely used in pregnancy to treat gestational diabetes mellitus and polycystic ovary syndrome (PCOS). Association between PCOS and developmental delay in offspring, and larger head circumference of metformin-exposed newborns has been reported. The objective of this study was to explore whether metformin exposure in utero had any effect on offspring cognitive function.

**Method:**

The current study is a follow-up of two randomized, placebo-controlled studies which were conducted at 11 public hospitals in Norway In the baseline studies (conducted in 2000–2003, and 2005–2009), participants were randomized to metformin 1700 and 2000 mg/d or placebo from first trimester to delivery. There was no intervention in the current study. We invited parents of 292 children to give permission for their children to participate; 93 children were included (mean age 7.7 years). The follow-up study was conducted in 2014–2016. The Wechsler Preschool and Primary Scale of Intelligence version III and the Wechsler Intelligence Scale for Children version IV were applied for cognitive assessment. Androstenedione and testosterone were measured in maternal blood samples at four time-points in pregnancy.

**Results:**

We found no difference in mean, full scale IQ in metformin (100.0 (SD 13.2)) vs. placebo-exposed (100.9 (SD 10.1)) children. There was an association between metformin exposure in utero and borderline intellectual function of children (full scale IQ between 70 and 85). Free testosterone index in gestational week 19, and androstenedione in gestational week 36 correlated positively to full scale IQ.

**Conclusions:**

We found no evidence of long-term effect of metformin on average child cognitive function. The increase of borderline intellectual functioning in metformin-exposed children must be interpreted with caution due to small sample size.

**Trial registration:**

The baseline study was registered on 12 September 2005 at the US National Institute of Health (ClinicalTrials.gov) # NCT00159536.

## Background

Metformin is an old, low cost, oral antidiabetic drug used to treat type 2 diabetes mellitus, gestational diabetes mellitus (GDM) and polycystic ovary syndrome (PCOS). Metformin passes the placental barrier, thereby potentially affecting the foetus [1]. A recent large exploratory case-control study on metformin-use in first trimester of pregnancy found no evidence of teratogenicity [2].

During the last four decades, prescription of metformin in pregnancy has increased, reflecting the increased prevalence of overweight, obesity and GDM [3]. Women with PCOS have increased risk of pregnancy complications, such as GDM, preterm delivery, preeclampsia, hypertension, and small for gestational age babies [4–9]. Our research group has recently reported on the reduced incidence of late miscarriage and preterm deliveries in an individual patient data meta-analysis of three metformin vs. placebo-controlled RCTs: the Pilot study, the PregMet study and the PregMet 2 study [10–12]. Interestingly, in all three studies, metformin exposed offspring had a larger head, both at gestational age 32 weeks and at birth [13]. Follow-up of the children from the PregMet study also showed that metformin-exposed children at 4 and 8 years og age, had higher BMI, waist circumference, waist-to-height ratio and prevalence of obesity than those exposed to placebo [14, 15].

Recent research suggests higher risk of developmental delay and neurodevelopmental disorders among children born to mothers with PCOS, due to potential exposure to a hyperandrogen environment and insulin resistance in utero [16–18]. Bell et al. found that maternal PCOS status was associated with developmental delay in offspring followed up through 3 years of age [16]. In a population-based study of register data in Sweden, Kosidou et al. found an increased risk of autism spectrum disorder and attention deficit/hyperactivity disorder (ADHD) in children of mothers diagnosed with PCOS, supporting the hypothesis of potential negative neurodevelopmental effects of a hyperandrogen environment in utero [17, 18].

In a follow-up of a Finnish RCT on pregnant women with GDM, Ijäs et al. reported no difference in motor, social and language development at 18 months of age between metformin- and insulin-exposed offspring [19]. Two studies have examined neurodevelopmental outcome in 2-year-old children born to mothers with GDM treated with metformin or insulin during pregnancy, reporting no difference between the two groups [20, 21]. In the present study, the primary aim was to explore the cognitive function of children born to mothers with PCOS, exposed to metformin vs. placebo in utero. The secondary aim of the study was to investigate associations between maternal androgen levels in pregnancy and subsequent cognitive function of children.

## Methods

The present study (CogMet study) is a follow-up study of children whose mothers participated in “the Pilot study” and “the PregMet study”. Both were randomized, placebo-controlled, double-blinded studies, assessing the potential of metformin to reduce pregnancy complications in women with PCOS.

### The pilot study

Between 2000 and 2003, 40 women aged 18–40 with PCOS and with singleton pregnancies were included between 5 and 12 weeks of gestation at the Trondheim University Hospital. Eighteen were randomized to metformin (1700 mg daily) and 22 to placebo throughout pregnancy [10].

### The PregMet study

Between 2005 and 2009, 257 pregnant women aged 18–45 years with PCOS according to the Rotterdam criteria [22] were included with 274 singleton pregnancies at 5–12 weeks of gestation, at 11 study centers in Norway. Seventeen women participated twice. Participants were randomized to metformin (2000 mg daily) or placebo throughout pregnancy, and 80% had a self-reported intake of > 85% of the tablets [11].

### The CogMet study

**Fig. 1:**
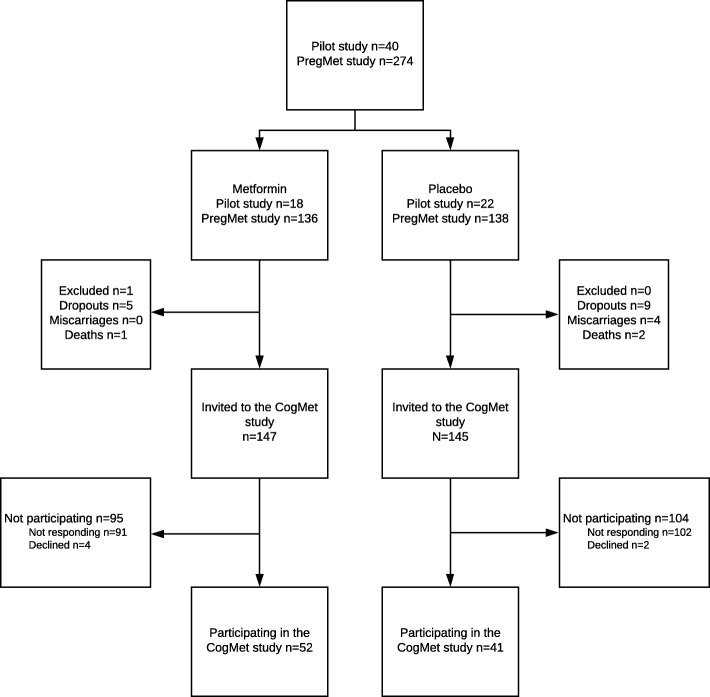
Flow-chart of inclusion and randomization to either metformin or placebo in the baseline studies (PregMet study and Pilot study), and the present study (Cogmet study)

In all, 292 children were eligible, and parents were invited to allow their children to participate in a follow-up with cognitive testing; 37 children from the Pilot study and 255 children from the PregMet study (Fig. 1). A total of 93 children were tested between April 2014 to June 2016, 52 in the metformin group, and 41 in the placebo group. The staff collecting the data and performing the cognitive testing were blinded to original randomization allocation. Information on gender, age and socio-economic status was obtained by standardized interviewer-administered questionnaires. Weight was measured with a digital weighing scale. Height was measured with a stadiometer (Seca, Germany). Head circumference was measured with a measuring tape over the most prominent part of the occiput, and just above the supraorbital ridge.

#### Cognitive assessment

Intelligence quotient (IQ) is a measure of cognitive function. Intellectual disability is considered to be a score on standardized intelligence tests that is more than two standard deviations below the population mean in combination with reported difficulties in daily life functioning, according the Diagnostic and Statistical Manual of Mental Disorders (DSM-V) [23]. This equals an IQ-score below 70. An IQ score within the normal range can be defined as a score within one standard deviation (SD) of the population mean (100), between 85 and 115 points. However, scores just below the normal range have been described as Borderline Intellectual Function in the DSM (IQ scores between approximately 70–85) and could also affect the individual’s ability to function in daily life [24].

The cognitive assessment was performed by three students specializing in clinical psychology, under the supervision of a clinical psychologist (HFØ) trained in cognitive testing. Testing took place during a single session with a fixed order of tasks. Age-appropriate Wechsler tests of cognitive ability were used for assessing full scale IQ (FIQ), IQ indices and subtest scores. The Wechsler tests are considered the gold standard in assessing intelligence, where different versions exist for preschool and school aged children. The Wechsler Preschool and Primary Scale of Intelligence version III (WPPSI-III) was applied for children under the age of six, and the Wechsler Intelligence Scale for Children version IV (WISC-IV) was applied for children above the age of six.

The WISC-IV consists of 15 subtests divided into scales that provide Verbal IQ and Performance IQ, respectively, in addition to FIQ. The verbal subtests are further divided into two indices: Verbal Comprehension and Working Memory. The performance scale tasks are divided into the Perceptual Organization Index and the Processing Speed Index. WPPSI-III consists of 14 subtests that are divided into the same scales and indices as the WISC-IV. The WPPSI-III and the WISC-IV group means are identical in the overlapping age-bands, and their respective FIQ scores are highly inter-correlated (i.e. r = 0.89) [25].

#### Androgen analyses

In the original RCTs maternal blood was drawn at the time of study inclusion, and in gestational weeks 19, 32 and 36. Dehydroepiandrosterone sulphate (DHEAS), sex hormone binding globulin (SHBG), and testosterone in maternal venous serum were analyzed. The DHEAS and SHBG levels were analyzed in single measurements by enzyme-linked immunosorbent assay techniques with the reagents and calibrators supplied by the manufacturer (DRG Instruments GmbH). Testosterone and androstenedione were analyzed by liquid chromatography-tandem mass spectrometry (LC-MS/MS). In the analysis, plasma samples were extracted by supported liquid extraction and the eluate evaporated and reconstituted before analysis on LC-MS/MS. The intra-assay and inter-assay coefficients of variation (CV) were 3.6 and 4.4% for DHEAS, and 2.9 and 11.4% for testosterone, respectively. The intra-assay CV was 6.6% for SHBG, and the inter-assay CV was 5.5% in fetal samples (lower values) and 24.1% in maternal samples. The free testosterone index (FTI) was calculated as (testosterone/SHBG) × 100 [26].

#### Statistical analysis

Data entry, management and analyses were performed at the Department of Clinical and Molecular Medicine at the Norwegian University of Science and Technology on IBM SPSS Statistics version 22.0 (IBM, SPSS inc USA, Chicago IL). Analyses were conducted using IBM SPSS Statistics version 25 (IBM, SPSS inc USA, Chicago IL). Differences between the groups were compared by independent t-test for continuous variables or the chi-square test and Fisher’s exact test for categorical variables. Linear regression analyses were used to compare continuous variables adjusted for parental education. Normality of residuals was confirmed by visual inspection of qq-plots. For calculation of anthropometric z-scores at birth, Niklasson’s standard values from a large Swedish population were applied [27]. The standard is gestational age adjusted and is based on singleton fetuses without chromosome abnormalities or major birth defects. At follow-up, z-scores for length/height, weight, and head circumference were computed from Norwegian growth references. These are built on anthropometric measurements of live-born children between 37 and 42 weeks of gestation, without congenital anomalies or diseases that could affect growth, and whose parents were from Norway or northern Europe [28]. Correlation coefficients ranging 0–0.29 were considered as small, 0.3–0.59 as moderate, and 0.6–1 as high. A significance level of 0.05 was chosen, and 95% confidence intervals (CI) are reported where relevant.

## Results

The response rate for the follow-up was 32% (93 out of 292). There was no difference between participants and non-participants regarding maternal baseline data, pregnancy outcomes and neonatal data (Additional file 1: Table S1). Maternal baseline data at original study entrance in early pregnancy, and pregnancy outcomes were comparable between the metformin and placebo groups (Table 1). There were no significant between-group differences in neonatal data, except larger head circumference and head circumference z-score of the newborns in the metformin group (Table 1). There was no difference in the prevalence of asthma, eczema, heart conditions, bowel disease, constipation, diarrhea, abnormal growth, recurrent infections or hospital admissions between the metformin vs. placebo exposed children. Parental education and socioeconomic status at follow-up were comparable between groups (Table 2). Mean age at follow-up was 7.7 years in both study groups (age range 5–14 years). All anthropometric measures, including Tanner stage development of the children at follow-up, were also comparable between groups (Table 2).

**Table 1:** Maternal baseline characteristics, pregnancy outcome and neonatal data

	Metformin (*n* = 52)	Placebo (*n* = 41)	*p*-value
**Baseline data of the mothers in 1st trimester**	*Mean (SD)*	*Mean (SD)*	
Age (years)	29.3 (3.4)	29.0 (4.3)	.762
Weight (kg)	84.7 (19.4)	79.5 (19.2)	.215
BMI^a^ (kg/m^2^)	30.2 (7.0)	27.9 (6.3)	.105
BP^b^_systolic_	121 (14)	117 (10)	.157
BP _diastolic_	75 (10)	74 (7)	.569
Phenotype PCOS^c^:	*n (%)*	*n (%)*	.397
A	26 (59)	21 (62)	
B	1 (2)	0	
C	5 (11)	1 (3)	
D	12 (27)	12 (35)	
Smoking	4 (8)	1 (3)	.279
**Pregnancy outcome**			
Gestational diabetes	12 (24)	11 (28)	.614
Preeclampsia	4 (8)	2 (5)	.609
Pre-term delivery	1 (2)	4 (10)	.089
	*Mean (SD)*	*Mean (SD)*	
Breastfeeding (months)	8.5 (4.5)	8.3 (3.8)	.864
**Adherence to study medication**^d^	*n (%)*	*n (%)*	.724
Good	42 (82)	30 (77)	
Acceptable	3 (6)	4 (10)	
Poor	6 (12)	5 (13)	
**Neonatal data**			
Gender male/female	22/28	17/23	.887
Apgar< 7 at 5 min	0	1 (3)	.252
	*Mean (SD)*	*Mean (SD)*	
Weight (g)	3475 (482)	3582 (793)	.491
Weight z-score	−0.28 (1.1)	0.07 (0.9)	.133
Length (cm)	49.8 (2.1)	49.7 (3.4)	.933
Length z-score	−0.71 (1.3)	− 0.49 (1.2)	.425
Head circumference (cm)	**35.7 (1.2)**	**34.7 (1.8)**	**.009**
Head circumference z-score	**0.50 (1.0)**	**−0.02 (1.0)**	**.022**
Gestational age (weeks)	39.1 (1.3)	38.7 (2.7)	.508

^a^ *BMI* body mass index, ^b^ *BP* blood pressure, ^c^ A: hyperandrogenism (HA) + oligoamenorrhea (OA) + polycystic ovaries (PCO), B: HA + OA, C: HA + PCO, D: OA + PCO. ^d^ good compliance: medication taken as intended; acceptable compliance: reduced medication to one to two tablets per day for a maximum of 4 weeks and/or no tablets for a maximum 2 weeks, otherwise as intended; poor compliance: reduced medication to one to two tablets per day more than 4 weeks and/or no tablets more than 2 weeks

**Table 2:** Socioeconomic status and offspring characteristics at follow-up

	Metformin (n = 52)	Placebo (n = 41)	*p*-value
**SES at follow-up**	*Mean (SD)*	*Mean (SD)*	
Education mother^a^	3.0 (0.8)	3.0 (0.9)	.910
	*n (%)*	*n (%)*	
10 years primary school	1 (2)	0	
high school	13 (28)	13 (37)	
less than 4 years of higher education	19 (40)	9 (26)	
4 or more years of higher education	14 (30)	13 (37)	
	*Mean (SD)*	*Mean (SD)*	
Education father^b^	2.7 (0.8)	2.6 (0.8)	.673
	*n (%)*	*n (%)*	
10 years primary school	0	2 (6)	
high school	26 (54)	15 (43)	
3 years of higher education	10 (21)	12 (34)	
5 or more years of higher education	12 (25)	6 (17)	
Parents’ relation:	*n (%)*	*n (%)*	.614
Married	27 (64)	22 (69)	
Cohabitant	11 (26)	6 (19)	
Divorced/separated	4 (10)	3 (9)	
Other	0	1 (3)	
**Offspring characteristics at follow-up**	*Mean (SD)*	*Mean (SD)*	
Age (years)	7.7 (2.7)	7.7 (2.6)	.958
Weight (kg)	34.3 (18.3)	30.8 (11.1)	.324
Weight z-score	0.60 (1.2)	0.31 (1.2)	.280
Height (cm)	131.7 (17.4)	130.2 (14.1)	.672
Height z-score	0.17 (0.9)	0.02 (1.1)	.491
BMI	18.7 (4.4)	17.6 (2.9)	.235
Head circumference (cm)	53.1 (2.2)	53.0 (1.9)	.804
Head circumference z-score	0.49 (1.5)	0.37 (1.3)	.696
Tanner stage^c^	1.5 (1.2)	1.4 (0.8)	.471

*SES* socioeconomic status

^a^ maternal education measured on a scale 1–4 (1:10 y primary school, 2: high school, 3: less than 4 years of higher education, 4: 4 or more years of higher education) ^b^ paternal education measured on a scale 1–4 (1: 10 y primary school, 2: high school, 3: 3 years of higher education, 4: 5 or more years of higher education) c: scale 1–5, 1 = prepubertal, 5 = adult size genitals and breast, adult pubic hair growth

The mean FIQ in the metformin and placebo groups were 100.0 (SD 13.2) and 100.9 (SD 10.1) respectively, which correspond to the average FIQ score in the background population. There was no statistically significant difference in mean FIQ scores between metformin and placebo exposed children at the follow-up (Table 3). Further, there was no statistically significant between-group difference in scores on the subscales: verbal comprehension, working memory, perceptual organization or processing speed (Table 3). These results did not change after adjustment for maternal/parental educational level (Table 4). None of the children had FIQ < 70 corresponding to intellectual disability. Ten children had FIQ < 85 corresponding to borderline intellectual function, 9 in the metformin and 1 in the placebo group, (*p* = 0.039). There were no significant differences regarding gender, prematurity, birth weight, head circumference or maternal baseline data between participants with borderline intellectual function and participants with normal intellectual function (data not shown). Baseline characteristics regarding maternal and neonatal data of children with borderline intellectual function are shown in Table 5.

**Table 3:** WPPSI-III/WISC-IV sum scores for metformin versus placebo, unadjusted

	Metformin (n = 52)	Placebo (n = 41)	p-value
WPPSI-III/WISC-IV score			
Full-scale IQ mean (SD)	100.0 (13.2)	100.9 (10.1)	.731
	*n (%)*	*n (%)*	**.039**
FIQ < 70	0	0	
FIQ 70–84	9 (17.3)	1 (2.4)	
FIQ > 84	43 (82.7)	40 (97.6)	
Indices			
Verbal comprehension (SD)	99.1 (15.6)	99.6 (14.3)	.887
Working memory (SD)	93.9 (14.8)	96.5 (10.8)	.323
Perceptual organization (SD)	107.3 (15.1)	108.2 (13.8)	.759
Processing speed (SD)	101.1 (19.7)	98.1 (12.9)	.397

*WPPSI-III* Wechsler Preschool and Primary Scale of Intelligence - Third edition

*WISC-IV* Wechsler Intelligence Scale for Children - Fourth edition

**Table 4:** Linear model of prediction of WPPSI-II/WISC-IV scores. Adjusted for parents’ educational level

		B (95% CI)	SE B	β	*p*-value	R^2^
Full scale IQ						0.10
	Metformin/placebo	1.6 (−3.6, 6.7)	2.59	0.55	.550	
	Mother’s education	4.6 (1.3, 8.0)	1.68	0.32	**.007**	
	Father’s education	−0.5 (−3.8, 2.9)	1.69	−0.03	.788	
Verbal comprehension						0.09
	Metformin/placebo	2.2 (−4.4, 8.7)	3.30	0.07	.513	
	Mother’s education	5.4 (1.1, 9.6)	2.14	0.30	**.014**	
	Father’s education	−0.4 (−4.7,3.8)	2.15	−0.03	.836	
Working memory						0.07
	Metformin/placebo	2.4 (−3.3, 8.2)	2.89	0.09	.404	
	Mother’s education	4.1 (0.4, 7.8)	1.87	0.26	**.030**	
	Father’s education	−0.5 (−4.2, 3.3)	1.88	−0.03	.802	
Perceptual organization						0.10
	Metformin/placebo	1.5 (−4.9, 7.9)	3.21	0.05	.640	
	Mother’s education	5.5 (1.4, 9.6)	2.08	0.31	**.010**	
	Father’s education	0.3 (−3.8, 4.5)	2.09	0.02	.873	
Processing speed						0.02
	Metformin/placebo	−3.2 (−11.1, 4.7)	3.97	−0.09	.419	
	Mother’s education	1.9 (−3.2, 7.0)	2.57	0.09	.469	
	Father’s education	−2.7 (−7.8, 2.4)	2.58	−0.13	.299	

*WPPSI-III* Wechsler Preschool and Primary Scale of Intelligence - Third edition

*WISC-IV* Wechsler Intelligence Scale for Children - Fourth edition

**Table 5:** Maternal and neonatal data, children with full-scale IQ < 85

	Number	Mean	Range
Baseline data of the mothers in 1st trimester			
Age (years)	10	27.9	21–35
Weight (kg)	10	81,9	65–106
BMI^a^ (kg/m^2^)	10	29.4	22–37
Pregnancy outcome			
Gestational diabetes	3/10		
Preeclampsia	0/10		
Pre-term delivery	0/10		
Neonatal data			
Gender male/female	3/7		
Apgar< 7 at 5 min	0/10		
Weight (kg)	10	3.5	3.1–4.2
Length (cm)	10	49.5	46–51
Head circumference (cm)	10	35.2	32–38
Gestational age (weeks)	10	39.3	37–41

^a^ *BMI* body mass index

Correlation between androgen levels at different time points in pregnancy and IQ-scores of children at follow-up, are shown in Table 6. There was a significant positive correlation between FIQ and maternal FTI measured at gestational week 19 (*p* = 0.041), and between androstenedione measured at gestational week 36 (*p* = 0.049). We found no correlation between maternal testosterone, androstenedione and FTI at any time of pregnancy in any subdomain scores of WISC-IV/WPPSI (Table 6). However, all correlation coefficients were small.

**Table 6:** Correlation between maternal androgen levels in pregnancy and child cognitive function measured by WPPSI-III/WISC-IV sum scores

	FIQ	VC	WM	PO	PS
Testosterone (T) at inclusion	.093	.117	−.084	.036	.076
T w 19	.223	.181	.038	.105	.162
T w 32	.205	.156	.144	.060	.158
T w 36	.218	.108	.158	.097	.200
Androstenedione (A) at inclusion	.010	.024	−.073	.001	.005
A w 19	.236	.155	.063	.130	.214
A w 32	.232	.163	.190	.104	.165
A w 36	**.255**	.157	.176	.124	.224
Free testosterone index (FTI) at inclusion	−.054	.004	−.096	−.087	−.042
FTI w 19	**.252**	.224	.129	.122	.148
FTI w 32	.228	.117	.166	.100	.156
FTI w 36	.197	.127	.159	.080	.158

*W* pregnancy week, *FIQ* full scale IQ, *VC* verbal comprehension, *WM* working memory, *PO* perceptual organization, *PS* processing speed. Statistically significant correlations are marked by bold numbers

## Discussion

The main finding of the present study is that metformin exposure in utero did not seem to affect mean cognitive function of children born to women with PCOS. To the best of our knowledge, this is the first follow-up of cognitive function of children exposed to metformin in utero in a placebo-controlled RCT.

The study question arose from the surprising finding of significantly larger newborn head circumference in the two RCTs, the Pilot and the PregMet studies [10, 13], which were reproduced in a third larger RCT, the PregMet2 study [12]. This led to the question of whether the larger head circumference in the metformin-exposed offspring translated to altered cognitive function in the offspring. On a group level, larger head circumference at birth is associated with better cognitive function [29, 30]. However, also autistic spectrum disorders associate positively with large head size [31]. In the general population the measures of cognitive function follow a normal distribution. The prevalence of intellectual disability (FIQ < 70) is about 1%, and intellectual disability is found to be associated with several disorders, such as autism spectrum disorders, ADHD, conduct disorders, epilepsy and sensory and motor impairments [32]. It is well established that cognitive function is dependent on both genetic and environmental factors [33, 34]. Among these are: sociocultural and family environment, education, nutrition, early stress, exposure to toxins, drugs or alcohol prenatally or in childhood, and perinatal complications [33, 34]. The potential identification of metformin as a factor that could alter cognitive function in prenatally exposed offspring is of great importance. While impaired cognitive function could represent social, behavioral and educational challenges for the individual and costs for society, good cognitive function is associated with beneficial outcome for the individual [32, 35]. Intelligence is considered a relatively stable measure throughout life [36]. In a Danish cohort-study, Flensborg-Madsen and colleagues found that birth weight and head circumference at birth was positively associated with intelligence in adulthood, and persisted into midlife [37].

At birth, metformin-exposed offspring in the CogMet study had significantly larger head circumference z-scores (Table 1). At the present follow-up, the difference in HC z-score was no longer significant (Table 2), and there was no statistically significant difference in mean IQ-scores of children in the two study groups (Table 3). However, exploring children with FIQ-score < 85, we found that there were more metformin-exposed children with borderline intellectual function compared to unexposed children. This is a surprising finding that should be interpreted with great caution due to small study size. We have no mechanistic/cellular biologic explanations for our findings and can only speculate. Metformin passes the blood-brain barrier and has been shown in experimental studies to have a neuroprotective effect in cerebral damage such as oxidative stress, blood-brain barrier disruption and inflammation [38]. Metformin has a mild and specific inhibitory effect on mitochondrial respiratory chain complex activity, switching the cell from anabolic to catabolic state [39]. Theoretically, this might affect the developing fetal brain.

Based on previous results of increased head circumference of metformin exposed newborns [13], and the results of Smithers et al. and Gale et al. of positive associations between head size and cognitive function [29, 30], we would have expected an opposite result. However, the total prevalence of individuals with FIQ < 85 in the study population, does not exceed the expected number based on an assumption of normal distribution of IQ scores. The FIQ scores had a significant positive correlation to maternal education in the metformin group.

Nonetheless, these findings point to the importance of investigating cognitive function in children exposed to metformin in utero. The secondary aim of the present study was to investigate associations between maternal androgen levels and cognitive function in children. Previous research indicates that a hyperandrogenic environment in utero might be associated with impaired development and neurodevelopmental disorders such as ADHD and autism spectrum disorder [16–18]. We found statistically significant, but small, positive correlations of FIQ and maternal FTI at gestational week 19, and androstenedione at gestational week 36. However, these were the only statistically significant correlations among a large number of tests, and should therefore be interpreted with caution. We did not find convincing evidence for an association between hyperandrogen environment in utero and cognitive function in children.

### Strengths and limitations

The well characterized cohort of women with PCOS and the randomized, placebo-controlled study design is a strength of the study. This is the first long-term follow-up on exposure of metformin versus placebo in utero of children including cognitive assessment, thereby an important contribution to the present base of knowledge regarding long-term consequences of metformin use in pregnancy. The main limitation of the present study is the relatively low number of participants, and a low response rate of only 32% of those invited. However, those who participated in the CogMet study did not differ at baseline from those who were invited, but chose not to participate (Additional file 1: Table S1). Particularly, there is a possibility that some children with low-to-borderline intellectual function were lost to follow-up due to the total burden of challenges these children and their families experience.

## Conclusion

Previously reported larger head circumference at birth in metformin-exposed children did not translate into altered mean cognitive function at a follow-up at 7.7 years (mean age). However, more of the metformin exposed children had borderline cognitive function, which is an unexpected and unexplained finding. We found no convincing evidence of an association between a high maternal androgen level and altered offspring cognitive function. Our findings are important contributions to the current knowledge of long-term consequences of metformin use during pregnancy. More research on larger study populations is needed.

## Supplementary information

**Additional file 1 : Table S1.** Baseline data from the original PregMet study, according to participation vs. non-participation in the present follow-up study.

## Data Availability

The datasets used and/or analysed during the current study are available from the corresponding author on reasonable request.

## Abbreviations

BMI: Body mass index
CI: Confidence interval
CV: Coefficients of variation
DHEAS: Dehydroepiandrosterone sulphate
DSM: Diagnostic and statistical manual of mental disorders
FIQ: Full-scale intelligence quotient
FTI: Free testosterone index
GDM: Gestational diabetes mellitus
IQ: Intelligence quotient
PCOS: Polycystic ovary syndrome
RCT: Randomized controlled trial
SD: Standard deviation
SHBG: Sex hormone binding globulin
WISC-IV: Wechsler Intelligence Scale for Children version IV
WPPSI-III: Wechsler Preschool and Primary Scale of Intelligence version III
