# Supplementary material for: Cognitive function in metformin exposed children, born to mothers with PCOS – follow-up of an RCT
Source: BMC Pediatr. 2020 Feb 10;20:60. doi: 10.1186/s12887-020-1960-2 (PMC7008569; doi:10.1186/s12887-020-1960-2)
Supplement: Supplementary file 1 — Additional file 1 : Table S1. Baseline data from the original PregMet study, according to participation vs. non-participation in the present follow-up study. [file 12887_2020_1960_MOESM1_ESM.docx]

**Table S1**

Baseline data from the original PregMet study, according to participation vs. non-participation in the present follow-up study.

|  | Participants  (n= 90) | Non-participants (n= 201) | p-value |
| --- | --- | --- | --- |
| **Baseline data of the mothers in 1^st^ trimester** | *Mean (SD)* | *Mean (SD)* |  |
| Age (years) | 29.2 (3.8) | 29.7 (4.6) | .352 |
| Weight (kg) | 82.5 (19.4) | 80.6 (19.3) | .456 |
| BMI^a^ (kg/m^2^) | 29.2 (6.8) | 28.9 (6.8) | .698 |
| BP^b^ _systolic_ | 119 (13) | 117 (11) | .215 |
| BP _diastolic_ | 74 (9) | 73 (12) | .397 |
|  |  |  |  |
| Phenotype PCOS^c^: | *n (%)* | *n (%)* | .297 |
| A | 47 (60) | 104 (58) |  |
| B | 1 (1) | 10 (6) |  |
| C | 6 (8) | 20 (11) |  |
| D | 24 (31) | 45 (25) |  |
| Smoking, n (%): | 5 (6) | 19 (10) | .194 |
|  |  |  |  |
| **Pregnancy outcome** |  |  |  |
| Gestational diabetes | 23 (26) | 50 (27) | .815 |
| Preeclampsia | 6 (7) | 10 (5) | .667 |
| Pre-term delivery | 5 (6) | 10 (5) | .951 |
|  | *Mean (SD)* | *Mean (SD)* |  |
|  |  |  |  |
| **Neonatal data** |  |  |  |
| Gender male/female | 39/51 | 97/87 | .145 |
| Apgar<7 at 5 min, n (%) | 1 (1) | 3 (2) | .815 |
|  | *Mean (SD)* | *Mean (SD)* |  |
| Weight (g) | 3522 (634) | 3573 (553) | .515 |
| Weight z-score | -0.05 (1.08) | 0.04 (1.05) | .516 |
| Length (cm) | 49.7 (2.7) | 50.5 (3.8) | .129 |
| Length z-score | -0.60 (1.19) | -0.39 (1.10) | .181 |
| Head circumference (cm) | 35.3 (1.6) | 35.3 (1.6) | .679 |
| Head circumference z-score | 0.20 (1.00) | 0.14 (1.10) | .688 |
| Gestational age (weeks) | 38.9 (2.0) | 39.2 (1.7) | .250 |
|  |  |  |  |
|  |  |  |  |

*Note.* a: BMI: body mass index, b: BP: blood pressure, c: A: hyperandrogenism (HA)+oligoamenorrhea (OA)+polycystic ovaries (PCO), B: HA+OA, C: HA+PCO, D: OA+PCO
